# Sex-Based Differences in Melanoma Survival Improvement from 2004 to 2018

**DOI:** 10.3390/cancers16071308

**Published:** 2024-03-27

**Authors:** Vikram R. Shaw, Angela Hudock, Baoyi Zhang, Christopher Amos, Chao Cheng

**Affiliations:** 1 Institute for Clinical and Translational Research, Baylor College of Medicine, Houston, TX 77030, USA; vikram.shaw@bcm.edu (V.R.S.); chris.amos@bcm.edu (C.A.); 2 School of Medicine, Baylor College of Medicine, Houston, TX 77030, USA; angela.hudock@bcm.edu; 3 Department of Chemical and Biomolecular Engineering, Rice University, Houston, TX 77005, USA; bz26@rice.edu; 4 Section of Epidemiology and Population Sciences, Department of Medicine, Baylor College of Medicine, Houston, TX 77030, USA; 5 Dan L Duncan Comprehensive Cancer Center, Baylor College of Medicine, Houston, TX 77030, USA

**Keywords:** melanoma, epidemiology, sex, cancer-specific survival, racial disparities in cancer survival, socioeconomic disparities in cancer survival

## Abstract

**Simple Summary:**

Melanoma is the deadliest form of skin cancer and its incidence and mortality may vary by demographic factors, such as sex, age, race, and socioeconomic status. Few studies, however, have characterized disparities in survival improvement across these demographic groups in melanoma. In the present study, the authors highlight disparities in melanoma survival improvement, both in diagnosed melanoma and carcinoma in situ. While melanoma survival has improved overall, some patient subgroups have experienced a lower improvement in survival from 2004 to 2018.

**Abstract:**

**Background:** Melanoma is the deadliest form of skin cancer and its incidence and mortality vary by sex, age, race, and socioeconomic status. Relatively few studies, however, have characterized disparities in survival improvement across these demographic groups in melanoma. Methods: Survival data from the Surveillance, Epidemiology, and End Results (SEER) database were obtained from 2004 to 2018. The compiled data were analyzed for cancer-specific survival (CSS) to produce multivariable Cox regressions that estimate sex-based survival disparities across patient demographic groups. Additionally, time-to-progression and survival analyses were conducted for a cohort of patients with carcinoma-in situ (CIS) that developed into melanoma. Results: In both female and male patients, melanoma diagnosis in more recent years (2014–2018 versus 2004–2008) was associated with an improved CSS, with females demonstrating an HR of 0.55 (95% CI: 0.49–0.60) and males demonstrating an HR of 0.49 (0.46–0.53). The trend remained consistent upon analyzing the effects of both sex and race on survival improvement for White and Hispanic males and females, but the results were not significant for Black and Asian patients. Joint sex and age analysis demonstrated significant reductions in HR across all age groups for female and male patients with a diagnosis in more recent years. Analysis of lesions progressing from CIS to melanoma (high-risk CIS) demonstrated an increased OR for males over females (OR: 1.70; 95% CI: 1.55–1.85), while survival analysis demonstrated no difference between sexes in the HR. Finally, for male patients, high-risk CIS demonstrated worse CSS compared to female patients with high-risk CIS (OR: 1.43; 95% CI: 1.15–1.79). Conclusion: Overall, melanoma survival has improved in recent years, though some patient subgroups have experienced a lower improvement in survival from 2004 to 2018.

## 1. Introduction

Melanoma is the deadliest form of skin cancer, resulting in approximately 75% of skin cancer-related deaths annually [1]. Furthermore, the incidence of melanoma continues to rise, representing a major public health risk [1]. Recent improvements in oncological care, however, such as the implementation of novel biologic treatments, have resulted in increases in melanoma survival [2].

It is known that differences in treatment outcomes for different race, sex, age, and socioeconomic groups are strongly influenced by the ability to access healthcare equitably across all groups [1,3,4,5]. Being aware of where these disparities lie may allow clinicians to identify the most vulnerable groups and understand where the largest gains in equitable care can be made [1,4,5]. Historically, melanoma has been considered a disease primarily of patients with lighter-pigmented skin and the largest improvements in cancer-specific survival (CSS) have been observed in this population [4]. The recent literature on melanoma care has demonstrated the presence of disparities in outcomes for different racial and socioeconomic groups, while others have explored the impact of socioeconomic status on diagnosis and CSS [1,4]. However, to our knowledge, a gap in the literature exists that considers sex in combination with other demographic factors. The aim of this study is to determine how sex, race, age, cancer stage, and income can affect the cancer-specific survival (CSS) of melanoma and specifically how melanoma survival improvement from 2004 to 2018 may vary among sexes within these groups.

The present analysis shows that lower income, being a racial minority, advanced age, advanced stage, and male sex are all associated with an increased hazards ratio (HR) in melanoma. Regardless of patient sex, decreases in HR were associated with diagnosis in more recent years. White female patients showed the largest decreases in HR over time, followed by Hispanic female patients who demonstrated significant decreases only in the 2014–2018 time period. There were no significant changes in HR for Black and Asian female patients with progressing time. Among male patients, White male patients showed the largest decrease in HR in the 2009–2013 period and Hispanic male patients showed the largest decrease from 2014 to 2018. There were no significant changes for Black and Asian male patients with progressing time.

## 2. Methods

### 2.1. SEER Database

The Surveillance, Epidemiology, and End Results (SEER) database is a population-based cancer registry. The SEER 18 registries Incidence-Based Mortality data from 2004 to 2018 for cancer patients were analyzed in the present study and the following selection criteria were used: (1) “Type of Reporting Source” is “Hospital inpatient/outpatient or clinic”, (2) patient diagnosed with only one primary cancer as indicated by “One primary only” in the variable “Sequence number” provided by SEER, and (3) age of diagnosis between 40 and 85.

Overall and cancer-specific survival information was determined based on the following variables: “Vital status recode (study cutoff used)”, “SEER cause-specific death classification”, and “Survival months”. Demographic information was also considered, and racial and ethnic data were determined using the following variables: “Race and origin recode (NHW, NHB, NHAIAN, NHAPI, Hispanic)”, from which “Non-Hispanic White”, “Non-Hispanic Black”, “Non-Hispanic Asian or Pacific Islander”, and “Hispanic (All Races)” were categorized as White, Black, Asian, and “Hispanic”. Age, sex, and annual income information were determined based on the following variables, respectively: “Age recode with single ages and 85+”, “Sex”, and “Median household income inflation adj to 2019”. The cancer stage was determined based on “Derived AJCC Stage Group, 6th ed (2004–2015)”, “Derived SEER Cmb Stg Grp (2016–2017)”, and “Derived EOD 2018 Stage Group (2018+)” for patients diagnosed during 2004–2015, 2016–2017, and 2018, respectively.

For the carcinoma in situ (CIS) analysis, the non-progressive CIS cohort was defined by patients with only one record of a melanoma CIS. The multiple CIS cohort was defined by patients with a CIS record followed by a second diagnosis of CIS in a later year. The high-risk CIS cohort was defined by patients with one or multiple CIS records, followed by a diagnosis of melanoma in a later year. Of note, the melanoma that occurred may not have arisen from the initial CIS diagnosis.

### 2.2. Survival and Statistical Analysis

Survival analysis for cancer-specific survival (CSS) and time to progression was performed with the R package “survival”. [6] Multivariable Cox regression utilizing the Efron approximation was used to estimate sex-based survival disparities in different patient subgroups, conditioned on race, age (40–55, Younger; 56–70, Middle; 71–85, Older), and income (<USD 60,000, Low; USD 60,000–USD 74,999, Intermediate; >USD 75,000, High). The income groups were selected to include groups both above and below the US median income of ~USD 70,000. Survival improvement disparities were investigated by grouping patients into three 5-year bins based on their year of diagnosis, as follows: 2004–2008 (reference group), 2009–2013, and 2014–2018. Multivariable Cox regression was used to calculate the hazard ratio (HR) and 95% confidence intervals (CIs) for each 5-year survival period in different patient subgroups. *p*-values less than 0.05 were considered significant in the present analysis. A saturated Cox model with interaction terms was also constructed, including pairwise interaction terms between sex and race, stage, income, age at diagnosis, and year of diagnosis. For the CIS analysis, the linear model for progression included sex, diagnosis year, age, marital status, prior site, diagnosis at prior site, laterality, income, rural status, and surgery status as explanatory variables for progression. The survival model included the same variables, except the outcome variable was instead time to progression. Finally, the cancer-specific survival Cox regression model for the CIS analysis included sex, age, year of diagnosis, and stage as explanatory variables for time to cancer-related death. 

## 3. Results

### 3.1. Cancer-Specific Survival by Demographic Categories

**Figure 1:**
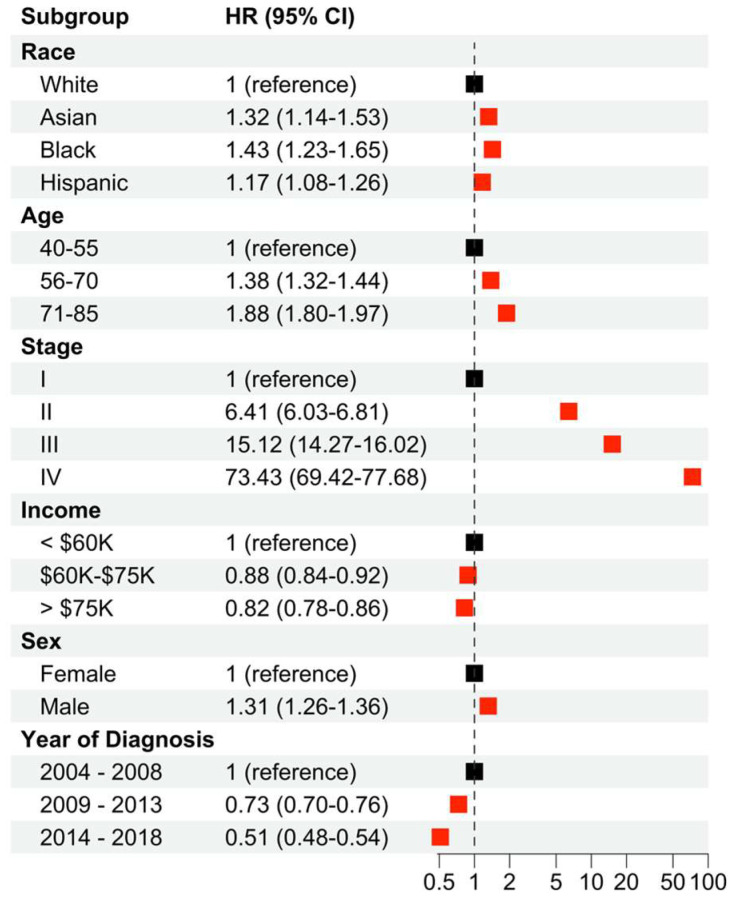
Multivariable Cox regression analysis to determine the effects of race, age at diagnosis, stage, income, and sex on CSS for melanoma. Hazard ratios and 95% confidence intervals reported for each of the measured groups. The baseline hazard is White, age 40–55, Stage I, income < USD 60 K, female, and year of diagnosis from 2004 to 2008. Red squares indicate significance (*p* < 0.05) while black squares are not significant.

Cancer-specific survival (CSS), or melanoma-specific survival in the present study, was investigated to determine the effects of race, age, stage, income, sex, and year of diagnosis on melanoma outcomes (Figure 1, Supplementary Table S1). Compared to white patients, Asian, Black, and Hispanic patients demonstrated a significantly higher hazard ratio (HR), suggesting worse survival. In particular, Black patients demonstrated the highest HR of 1.43 (95% CI: 1.23–1.65). Both age and stage demonstrated an incremental relationship, with each stepwise increase in age at diagnosis leading to an increased HR. The oldest age group (71–85 years old) demonstrated an HR of 1.88 (95% CI: 1.80–1.97) and stage IV patients demonstrated an HR of 73.43 (95% CI: 69.42–77.68). Higher income levels were associated with a decreased HR for both the middle- and high-income groups, compared to the low-income group. Finally, male patients demonstrated an increased HR of 1.31 (95% CI: 1.26–1.36) when compared to female patients. In a saturated model including pairwise interaction terms between sex and race, stage, income, age at diagnosis, and year of diagnosis (Supplementary Table S2), notable significant interaction terms included Black and male, Stage II and male, Stage III and male, and Stage IV and male.

### 3.2. Sex-Based Analysis of Melanoma Survival Improvement from 2004 to 2018

**Figure 2:**
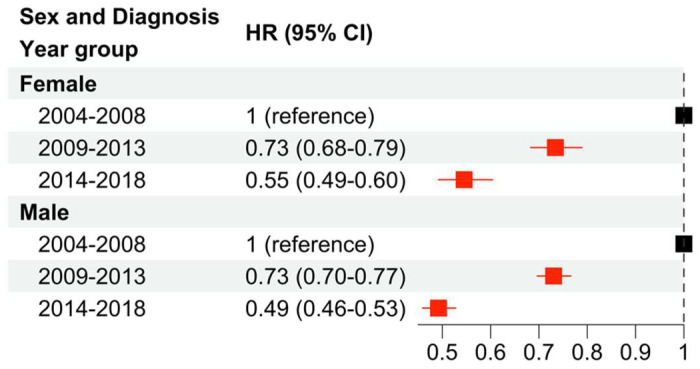
Multivariable Cox regression analysis to examine changes in CSS due to sex and year of diagnosis. The baseline hazard is diagnosis from 2004 to 2008. Red squares indicate significance (*p* < 0.05) while black squares are not significant.

Subsequently, multivariable Cox regression analysis was performed to examine changes in CSS stratified by sex from 2004 to 2018 (Figure 2). Across both females and males, CSS improved in more recent years compared to the reference time period (2004–2008). Additionally, similar improvements were demonstrated across both females and males across each age bin comparison (i.e., 2009–2013 versus 2004–2008 and 2014–2018 versus 2004–2008).

### 3.3. Sex-Based Differences in Melanoma Survival Improvement by Race from 2004 to 2018 

**Figure 3:**
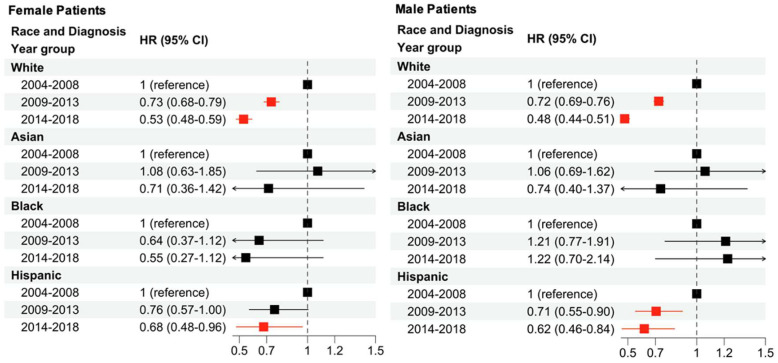
Multivariable Cox regression analysis to examine changes in CSS due to sex, race/ethnicity, and year of diagnosis. The baseline hazard is a diagnosis from 2004 to 2008, stratified by race and sex. Arrows indicate that the full confidence interval is not shown in the graphical depiction of the HR and 95% CI. Red squares indicate significance (*p* < 0.05) while black squares are not significant.

Subsequently, we investigated sex-based differences in survival improvement by race from 2004 to 2018 (Figure 3). In both White and Hispanic female and male patients, CSS improved in a stepwise manner during each subsequent time period. However, in Asian and Black patients, no improvement in CSS was observed. Additionally, while the differences were not significant, a lower HR was observed for Black females in the latter two time periods, while an increased HR was observed for Black males in the latter two time periods. Hispanic males demonstrated a significantly improved CSS across both latter time periods, while only the most recent time period demonstrated an improved CSS for Hispanic females.

### 3.4. Sex-Based Similarities in Melanoma Survival Improvement by Age and Stage from 2004 to 2018

**Figure 4:**
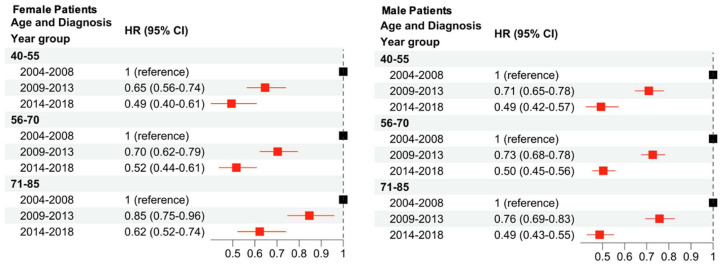
Multivariable Cox regression analysis to examine changes in CSS due to sex, age at diagnosis, and year of diagnosis. The baseline hazard is diagnosis between 2004 and 2008, stratified by age at diagnosis and sex. Red squares indicate significance (*p* < 0.05) while black squares are not significant.

**Figure 5:**
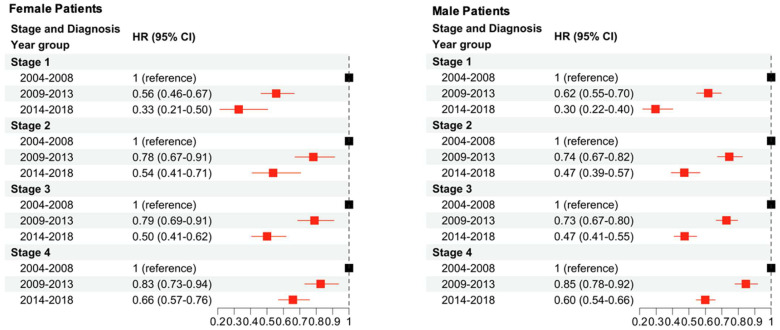
Multivariable Cox regression analysis to examine changes in CSS due to sex, stage, and year of diagnosis. The baseline hazard is diagnosis between 2004 and 2008, stratified by stage and sex. Red squares indicate significance (*p* < 0.05) while black squares are not significant.

The impact of sex and age at diagnosis on survival improvement was investigated (Figure 4). In both female and male patients, a consistent stepwise trend was seen across all age groups with an improvement in CSS in the latter two time periods. Subsequently, the impact of sex and stage on survival improvement was investigated (Figure 5). In both female and male patients, an improvement was seen in CSS, with the most significant improvement occurring across both sexes for patients diagnosed with stage I melanoma between 2014 and 2018. Across the stages and comparing the group diagnosed in 2014–2018 versus 2004–2008, stage IV patients demonstrated the smallest improvement in CSS.

### 3.5. Male Patients Associated with High-Risk CIS with Worse CSS

As described in the “Methods”, three cohorts of patients with CIS were studied, as follows: patients with non-progressive CIS, patients with multiple CIS, and patients with an initial diagnosis of CIS with a later diagnosis of melanoma (i.e., high-risk CIS) (Table 1). In both the multiple CIS and high-risk CIS versus non-progressive CIS groups, males demonstrated increased adjusted odds ratios (OR) of 1.30 (95% CI: 1.21–1.39) and 1.70 (95% CI: 1.55–1.85), respectively (Table 2). Survival analysis measuring time-to-progression demonstrated no significant sex-based differences in the multivariable Cox regression model. Finally, the analysis of CSS in patients with high-risk CIS versus non-progressive CIS demonstrated that males had a higher HR (1.43; 95% CI: 1.15–1.79).

## 4. Discussion

The present study aims to characterize the impacts of race, age, sex, economic status, and stage on CSS, as well as the compounding effects of sex and race or sex and age on CSS. It reinforces the current melanoma literature, which shows disparities in outcomes for minority groups and patients of lower socioeconomic status [3,4,5,7]. Particularly, the present study shows that a lower HR is found in patients who are White (as opposed to Black, Asian, or Hispanic), middle or high income (as opposed to low), female (as opposed to male), diagnosed during an earlier stage (as opposed to later stages), and younger when diagnosed (as opposed to older). Both male and female patients saw an increased CSS from 2004 to 2018, consistent with improvements in melanoma survival in recent years [2]. Additionally, this study demonstrates that the compounding of multiple demographic factors can affect CSS. For example, disparities between the sexes within races is demonstrated for both White patients and Hispanic patients. Both White male and female patients saw decreases in HR from 2004 to 2018, with larger reductions seen in White female patients. Hispanic male patients saw decreases in HR from 2004 to 2018 as well, but Hispanic female patients only saw a significant reduction in HR during the 2014–2018 period. When comparing age and sex, a significant decrease in HR over time was observed for all age groups and both sexes.

The findings of this paper also emphasize outcome disparities by socioeconomic status. In patients with a low socioeconomic status, challenges like access to care, distance to care, health literacy, medical mistrust, insurance status, and more may worsen outcomes [5]. While the overall CSS of melanoma is improving, the largest improvements are seen in those of higher socioeconomic status [1,4,7,8]. Examining the present data may highlight the current largest gaps to equitable care, such as the increase (though not significant) in HR seen in Black male patients from 2004 to 2018. This is one of the only groups analyzed that saw a trend of decreased CSS over time. Additionally, a significant positive interaction coefficient was observed among Black and male explanatory variables in the saturated model, suggesting that the combination of these factors may worsen melanoma outcomes. The literature suggests that Black and Hispanic patients may experience worse outcomes due to the underdiagnosis of melanoma in patients with darker pigmented skin until it is in later stages, which is associated with a worse prognosis [1,4,9]. Another hypothesis is the association of melanoma as a skin disease for light-skinned patients, which may decrease the likelihood of Black and Hispanic patients to seek regular skin checks due to lower perceived risk [1,5,9].

Previous studies have reported that melanoma survival is the highest in patients who are White, have higher incomes, or are diagnosed at earlier stages [1,4]. Patients with higher incomes experience improved outcomes for several potential reasons, such as increased preventative care, as well as being insured, which allows for access to routine surveillance through skin checks [1,5,9]. Regular skin checks can identify melanoma in the earlier stages, which can lead to better outcomes for the patient. This may explain the improvement in HR for male and female patients diagnosed with early stage (i.e., stage I and II) melanoma. Another possible reason for the observed trend is that providers may have a higher index of suspicion for melanoma in lighter-skinned patients [1,4,9]. Additionally, melanic lesions and nevi may present with greater contrast in patients with lighter skin, causing providers to notice and diagnose melanoma earlier [1,4,9]. In addition to the survival improvement in early-stage melanoma, patients with later-stage (i.e., stage III and IV) melanoma have also seen improvements in survival. This improvement is likely a result of recent advances in immunotherapy treatment options [10]. The median survival of patients with advanced, inoperable stage IV melanoma has improved from around 6 months to nearly 6 years, largely as a result of the improvements in therapy [10].

Previous studies have also shown the association of lower CSS with lower socioeconomic status [1,4,7]. Most of these studies, however, focused on a single time period, while the present study considers a stepwise timeline of three periods to examine the trends of data over time. This approach provides us with potential indications as to which groups may benefit the most from oncological treatment advancements, screening, and community outreach. Identifying the patient subgroups at the highest risk of decreased CSS may allow for targeted outreach and increased screening, in an attempt to close the risk gap between groups. 

Both sexes demonstrated decreases in HR in a stepwise fashion over the time periods investigated. For females, the youngest age group (40–55) saw larger decreases than the oldest age group, though this trend was not a significant difference. For males, the HR decrease was similar across all age groups and there did not appear to be any trends associated with patient age at time of diagnosis. In previous studies, the difference between the prognosis of melanoma for females and males has not been linked to presentation (ulcerated versus non-ulcerated), histological presentation, or Breslow thickness [11]. After controlling for all these variables, however, there is still a significant advantage in the female population for CSS [11]. This may be, in part, explained by the finding from our study that males have increased odds of multiple CIS and high-risk CIS, when compared to females. Additionally, our results demonstrate that CSS is worse in males with a high-risk CIS compared to females. Though the mechanism of the sex-based difference is not entirely clear, it has been found that localized melanoma is less likely to metastasize in female patients than in male patients, which could be an explanation for the decreased CSS in male patients [11]. Our findings are consistent with previously published work and support the importance of sex as a potential pre-specified variable in clinical trials [12].

In both the multiple CIS and high-risk CIS versus non-progressive CIS groups, males demonstrated increased adjusted odds ratios (OR) of 1.30 (95% CI: 1.21–1.39) and 1.70 (95% CI: 1.55–1.85), respectively (Table 2). Survival analysis measuring time-to-progression demonstrated no significant sex-based differences in the multivariable Cox regression model. Finally, the analysis of CSS in patients with high-risk CIS versus non-progressive CIS demonstrated that males had a higher HR (1.43; 95% CI: 1.15–1.79).

The present study has several limitations. One major limitation of this study is the relatively lower number of Black and Asian patients, as a larger sample size would have more definitively shown if the trends seen in this dataset were significant. Furthermore, large databases and population-based registries are subject to bias and errors, such as the presence of unrecorded variables, coding variability, reporting variability, patient migration between registry areas, early censoring, and missing data [13].

Future research in this area of inquiry is essential in understanding health disparities in melanoma survival among different demographic groups. The results of this study should be compared to the data from 2018 onward, as it becomes available, to identify how the trends may change. There is also an opportunity to study these effects in different types of skin cancer, or skin cancer as a whole, in addition to melanoma. Continuing to study multiple populations of patients diagnosed with melanoma and their outcomes will allow for a dynamic, informed, and equitable approach to treatment and prevention of melanoma.

## 5. Conclusions

The present analysis shows that lower income, being a racial minority, advanced age, advanced stage, and male sex are all associated with an increased hazards ratio (HR) in melanoma. Regardless of patient sex, decreases in HR were associated with diagnosis in more recent years. White female patients showed the largest decreases in HR over time, followed by Hispanic female patients who demonstrated significant decreases only in the 2014–2018 time period. There were no significant changes in HR for Black and Asian female patients with progressing time. Among male patients, White male patients showed the largest decrease in HR for 2009–2013 and Hispanic male patients showed the largest decrease from 2014 to 2018. There were no significant changes for Black and Asian male patients with progressing time. Finally, males demonstrated increased odds of progression from CIS to melanoma and worse CSS, compared to females with high-risk CIS.

## Figures and Tables

**Table 1:** Demographic characteristics of non-progressive carcinoma in situ (CIS), multiple CIS, and high-risk CIS. The non-progressive CIS cohort was defined by patients with only one record of a melanoma CIS. The multiple CIS cohort was defined by patients with a CIS record followed by a second diagnosis of CIS in a later year. The high-risk CIS cohort was defined by patients with one or multiple CIS records followed by a diagnosis of melanoma in a later year.

	Measure	Non-Progressive CIS (*n* = 57,680)	Multiple CIS (*n* = 7392)	High-Risk CIS (*n* = 5875)
Demographic characteristics	Sex (male, %)	29,910 (51.9)	4607 (62.3)	4010 (68.3)
	Age (years, %)			
	0–19	275 (0.5)	1 (<0.1)	3 (<0.1)
	20–39	6895 (12.0)	293 (6.4)	261 (4.4)
	40–59	20,998 (36.4)	2078 (45.1)	1597 (27.2)
	60–79	22,888 (39.7)	4200 (91.2)	3254 (55.4)
	80+	6555 (11.4)	820 (17.8)	760 (12.9)
	Unknown	69 (0.1)	—	—
	Year of diagnosis (Median, IQR)	2008 (2002–2012)	2008 (2001–2013)	2006 (2000–2011)

**Table 2:** Progression analysis and cancer-specific survival analysis of multiple CIS and high-risk CIS versus non-progressive CIS, stratified by sex.

	Model	Measure	Multiple CIS versus Non-Progressive CIS	High-Risk CIS versus Non-Progressive CIS
Progression analysis	Linear model measuring association between sex and progression ^+^	Male (OR, 95% CI)	1.30 (1.21–1.39) *	1.70 (1.55–1.85) *
	Survival analysis measuring time to progression ^++^	Male (HR, 95% CI)	0.97 (0.91–1.04)	0.95 (0.87–1.04)
Cancer-specific survival analysis	Survival analysis measuring time to cancer-related death ^+++^	Male (HR, 95% CI)	--	1.43 (1.15–1.79) *

^+^ Linear model included sex, diagnosis year, age, marital status, prior site, diagnosis at prior site, laterality, income, rural status, and surgery status as explanatory variables for progression. ^++^ Survival model for time-to-progression included sex, diagnosis year, age, marital status, prior site, diagnosis at prior site, laterality, income, rural status, and surgery status as explanatory variables. ^+++^ Cancer-specific survival Cox regression model included sex, age, year of diagnosis, and stage as explanatory variables for time to cancer-related death. * *p* = or <0.001. Note: Females are the reference group for each of the comparisons.

## Data Availability

The datasets supporting the conclusions of this article are available in the Surveillance, Epidemiology, and End Results (SEER) repository [https://seer.cancer.gov/]. Accessed on 1 January 2022.

## Supplementary Materials

The following supporting information can be downloaded at: https://www.mdpi.com/article/10.3390/cancers16071308/s1, Table S1: Demographic information for patients included in CSS analysis (i.e., main text Figure 1, Figure 2, Figure 3, Figure 4 and Figure 5); Table S2: Multivariable Cox regression model for CSS analysis utilizing interaction terms.
