# Supplementary material for: Sex-Based Differences in Melanoma Survival Improvement from 2004 to 2018
Source: Cancers (Basel). 2024 Mar 27;16(7):1308. doi: 10.3390/cancers16071308 (PMC11011041; doi:10.3390/cancers16071308)
Supplement: Supplementary file 1 [file cancers-16-01308-s001.zip › cancers-2919300-supplementary.pdf]

Supplementary Materials

TITLE: Sex-based differences in melanoma survival improvement from 2004 to 2018

Vikram R. Shaw[1], Angela Hudock[2], Baoyi Zhang[1], Christopher I. Amos[1,3,4], Chao Cheng[1,4,*]

Affiliations:

1. Institute for Clinical and Translational Research, Baylor College of Medicine, Houston, TX 77030, United States.

2. School of Medicine, Baylor College of Medicine, Houston, TX 77030, United States.

3. Section of Epidemiology and Population Sciences, Department of Medicine, Baylor College of Medicine, Houston, TX 77030, United States.

4. Dan L Duncan Comprehensive Cancer Center, Baylor College of Medicine, Houston, TX 77030, United States.

*Corresponding author: Chao Cheng (chao.cheng@bcm.edu)

Keywords: melanoma, epidemiology, sex

Feature	Count	Univariate Cox P-values
Total number of patients	**93,946**	
Year diagnosis*, median (IQR)	2012 (2008 – 2016)	$< 2 \times 10^{-16}$
Age diagnosis*, median (IQR)	61 (52 – 70)	$< 2 \times 10^{-16}$
Sex*		
Female (%)	38,977 (41.5)	Referent
Male (%)	54,969 (58.5)	2.77×10^{-12}
Race/ethnicity*		
Asian	723 (0.7)	5.51×10^{-11}
Black	536 (0.6)	7.43×10^{-14}
Hispanic	3,695 (3.9)	3.78×10^{-4}
White	87,931 (93.6)	Referent
Other	1,061 (1.1)	
Income*		
< $60,000	24,467 (26.0)	Referent
$60,000 - $74,999	36,539 (38.9)	$< 2 \times 10^{-16}$
>$75,000	32,924 (35.0)	$< 2 \times 10^{-16}$
Unknown	16 (0.0)	
Stage*		
I	63,671 (67.8)	Referent
II	14,301 (15.2)	$< 2 \times 10^{-16}$
III	9,698 (10.3)	$< 2 \times 10^{-16}$
IV	6,276 (6.7)	$< 2 \times 10^{-16}$

Supplementary Table 1. Demographic information for patients included in CSS analysis (i.e., main text Figures 1 – 5).

* Wald test for Univariate Cox regression was = $p < 5 \times 10^{-16}$

Interaction Model Table

	HR	l95	h95	p
Asian	1.2762170	1.0120768	1.6092948	0.0392626
Black	1.1563814	0.9287891	1.4397433	0.1938291
Hispanic	1.2143042	1.0718307	1.3757160	0.0022934
Age 56-70	1.3578276	1.2525061	1.4720054	0.0000000
Age 71-85	2.0009550	1.8418177	2.1738422	0.0000000
Stage II	7.0190347	6.3012398	7.8185959	0.0000000
Stage III	17.6764087	15.9534033	19.5855028	0.0000000
Stage IV	92.6043441	83.9339961	102.1703356	0.0000000
$60,000 - $74,999	0.9196336	0.8505852	0.9942871	0.0353929
>$75,000	0.8686615	0.7991223	0.9442519	0.0009417
2009 - 2013	0.7351550	0.6849186	0.7890760	0.0000000
2014 - 2018	0.5518781	0.4994855	0.6097664	0.0000000
Male	1.7737277	1.5471934	2.0334303	0.0000000
Asian:Male	1.0403418	0.7718010	1.4023190	0.7951607
Black:Male	1.4500595	1.0838907	1.9399306	0.0123337
Hispanic:Male	0.9285448	0.7896152	1.0919184	0.3699630
Stage II:Male	0.8649988	0.7591653	0.9855864	0.0294060
Stage III:Male	0.7844888	0.6930199	0.8880302	0.0001244
Stage IV:Male	0.7082645	0.6292478	0.7972034	0.0000000
($60,000 - $74,999):Male	0.9433924	0.8593551	1.0356477	0.2208969
>$75,000:Male	0.9252813	0.8368813	1.0230190	0.1295799
Age 56-70:Male	1.0151788	0.9215360	1.1183372	0.7602947
Age71-85:Male	0.9091573	0.8218409	1.0057505	0.0645069
(2009 - 2013):Male	1.0006781	0.9197798	1.0886917	0.9874252
(2014 - 2018):Male	0.8994303	0.7982786	1.0133993	0.0816314

Supplementary Table 2. Multivariable Cox regression model for CSS analysis utilizing interaction terms. Model statement: coxph(Surv(Time, Event)~Race + Age + Stage + Income + Year_Diagnosis + Sex + Race*Sex + Stage*Sex + Income*Sex + Age*Sex + Year_Diagnosis*Sex)
